# The immune suppressive microenvironment of human gliomas depends on the accumulation of bone marrow-derived macrophages in the center of the lesion

**DOI:** 10.1186/s40425-019-0536-x

**Published:** 2019-02-27

**Authors:** Laura Pinton, Elena Masetto, Marina Vettore, Samantha Solito, Sara Magri, Marta D’Andolfi, Paola Del Bianco, Giovanna Lollo, Jean-Pierre Benoit, Hideho Okada, Aaron Diaz, Alessandro Della Puppa, Susanna Mandruzzato

**Affiliations:** 1 Veneto Institute of Oncology IOV – IRCCS, Padova, Italy; 2 Department of Surgery, Oncology and Gastroenterology, University of Padova, Via Gattamelata, 64 35128 Padova, Italy; 3 LUNAM Universite - Micro et Nanomedecines Biomimetiques, F-49933 Angers, France; 4 Univ Lyon, Université Claude Bernard Lyon 1, CNRS, LAGEP UMR 5007, F-69100, VILLEURBANNE, Lyon, France; 5 INSERM U1066/CNRS 6021 University of ANGERS, cedex 9, 49933 Angers, France; 6 Department of Neurological Surgery, University of California, San Francisco, CA USA; 7 Parker Institute for Cancer Immunotherapy, San Francisco, CA USA; 8 Neurosurgery Unit, Azienda Ospedaliera di Padova, Padova, Italy

**Keywords:** Innate immunity, Tumor microenvironment, Tumor immunology, Immunological tolerance, Brain cancer

## Abstract

**Background:**

Systemic and local immune suppression plays a significant role in glioma progression. Glioma microenvironment contains both brain-resident microglial cells (MG) and bone marrow-derived macrophages (BMDM), but the study of their functional and immune regulatory activity has been hampered until now by the lack of markers allowing a proper identification and isolation to collect pure populations.

**Methods:**

Myeloid and lymphoid infiltrate were characterized in grade II, III and IV gliomas by multicolor flow cytometry, along with the composition of the cell subsets of circulating myeloid cells. Macrophages were sorted and tested for their immunosuppressive ability. Moreover, following preoperative administration of 5-aminolevulinic acid to patients, distinct areas of tumor lesion were surgically removed and analyzed, based on protoporphyrin IX fluorescence emission.

**Results:**

The immune microenvironment of grade II to grade IV gliomas contains a large proportion of myeloid cells and a small proportion of lymphocytes expressing markers of dysfunctional activity. BMDM and resident MG cells were characterized through a combination of markers, thus permitting their geographical identification in the lesions, their sorting and subsequent analysis of the functional characteristics. The infiltration by BMDM reached the highest percentages in grade IV gliomas, and it increased from the periphery to the center of the lesion, where it exerted a strong immunosuppression that was, instead, absent in the marginal area. By contrast, MG showed little or no suppression. Functional differences, such as iron metabolism and phagocytosis, characterized resident versus blood-derived macrophages. Significant alterations in circulating monocytes were present in grade IV patients, correlating with accumulation of tumor macrophages.

**Conclusions:**

Grade IV gliomas have an alteration in both circulating and tumor-associated myeloid cells and, differently from grade II and III gliomas, show a significant presence of blood-derived, immune suppressive macrophages. BMDM and MG have different functional properties.

**Electronic supplementary material:**

The online version of this article (10.1186/s40425-019-0536-x) contains supplementary material, which is available to authorized users.

## Introduction

The concept of the immune privilege of the CNS has recently been revised and it appears now that local immunity can adapt to a peculiar environment, directed by a flexible blood brain barrier and by the presence of unconventional lymphatic vessels [1, 2]. Indeed, local immunity in the CNS is completely subverted by a growing tumor, as documented by the presence of a leukocyte infiltrate in different brain tumors [3]. Another peculiarity of the CNS is the presence of microglia (MG) cells, resident macrophages fulfilling the role of immune surveillance and removal of debris, with a distinct ontogenesis compared to bone-marrow derived macrophages (BMDM) that heavily infiltrate tumors [4, 5].

Primary brain tumors are heterogeneous not only in their genetic and metabolic composition, but also in their microenvironment. In glioblastoma (GBM), the presence and role of leukocyte infiltrating cells has been addressed in both mouse models and in human tumors. Elegant genetic mouse models have demonstrated that BMDM and MG are both present in gliomas and possess distinct transcriptional and chromatin states [6], and that during GBM growth there is an influx of myeloid cells in the tumor microenvironment [3, 7], which represents the main source of tumor-infiltrating macrophages. However, it is unclear to what extent a mouse model can recapitulate the human counterpart, given the heterogeneity of GBM. Also in grade II and III glioma patients, an infiltrate of myeloid origin mainly constituted of macrophages was documented [8, 9] and associated to shorter overall survival (OS) [10] or correlated to the pathological grade [11]. However, in all the studies performed in grade II to IV glioma patients, the precise identification of human MG cells from BMDM lacked or was limited to morphological evaluation coupled with immunohistochemical analysis [12], or to subtle differences in staining intensity of myeloid markers by flow cytometry, due to the lack of differentially expressed markers on the two cell types [7]. Recently, the addition of CD49D marker has been proposed to discriminate MG from BMDM [6, 10].

Given these constraints, the presence and relevance to tumor progression of BMDM and of resident MG is unclear in human gliomas. We sought to analyze the immune infiltrate in II, III and grade IV gliomas from freshly resected tissues, and to isolate and characterize MG from BMDM. Taking advantage of 5-aminolevulinic acid (5-ALA) administration to grade IV glioma (glioblastoma, GBM) patients prior to surgery, which leads to intracellular accumulation of fluorescent porphyrins [13], we analyzed separate areas of tumor lesions, from which we sorted both macrophage populations, thus enlightening their different immunological and functional characteristics.

## Methods

### Patient characteristics

Patients were recruited at the Department of Neurosurgery, Padova University Hospital, Italy and their characteristics are shown in Table 1. The ethical committee of the IOV-IRCCS and of Padova University Hospital approved all experiments and all patients gave their informed consent. The studies were conducted in accordance with the Declaration of Helsinki.

**Table 1:** Participant characteristics

	Glioma grade^a^			Meningioma^b^	Controls
	II	III	IV		
Sex (n)					
Male	7	8	54	3	24
Female	6	4	22	10	11
Median age	42	50	59	57	59
Range	24–70	29–73	27–79	43–74	36–84
IDH status(n)					
WT	1	3	72	NA	NA
Mutated	12	9	4	NA	NA
MGMT (n)					
Non-methylated	0	4	35	NA	NA
Methylated	6	4	38	NA	NA
NA	7	4	3	NA	NA
Steroid	no	yes	yes	yes	no

^a^For grade II, *n* = 13 patients and 13 tissue samples. For Grade III, *n* = 12 patients and 12 tissue samples. For Grade IV, *n* = 76 samples and 113 tissue samples

^b^Grade I and II

*n* = number

### Blood and tumor samples

Peripheral blood was drawn from patients either at surgery before anesthesia induction, or the day before surgery, and immediately processed. For functional assays peripheral blood mononuclear cells (PBMCs) were isolated by density gradient centrifugation on Ficoll-Paque PLUS (GE Healthcare-Amersham, NJ, USA), as previously described [14].

All tumors were processed immediately after resection by enzymatic digestion, using human Tumor Dissociation Kit (Miltenyi Biotec) and following manufacturer’s instructions for soft tumors. Before digestion, tissues were extensively washed with 0.9% sodium chloride solution to remove peripheral blood.

### Multiparametric flow cytometry

Peripheral blood was stained with monoclonal antibodies to analyze the presence of different myeloid cell populations. Staining procedure and immunophenotyping standardization were the same reported in [15] and are described in Additional file 1, containing the list of antibodies for cell subset analysis.

Cell suspension from glioma tissues after enzymatic digestion was labelled with different antibody mixtures to characterize myeloid and lymphocyte subsets as reported in Additional file 1: Table S1.

### Isolation of myeloid cell subsets and immunosuppressive assay

Live CD45^+^/CD33^high^**/**CD49D^+^/HLA-DR^+^ or live CD45^+^/CD33^high^/CD49D^−^/HLA-DR^+^ cell subsets were separated by FACS sorting (BD FACS ARIA III). The purity of each fraction was > 90%. Immunosuppressive activity of myeloid cells isolated either from the peripheral blood or from tumor, was performed as detailed in Additional file 1 and previously described [16].

### Cytospin preparation and may-Grünwald-Giemsa (MGG) staining

Sorted cells were centrifuged (Shandon Cytospin 3 centrifuge) on microscope slides, and cytospins were stained and analyzed as reported in [16].

### RNA-sequencing

CD11b^+^ cells were obtained by immunomagnetic cell sorting using anti-CD11b microbeads (Miltenyi Biotec), following manufacturer’s instruction, or by immunopanning. Single-cell RNA sequencing and data processing were performed as previously described [10].

### Statistics

The Mann-Whitney and the Student t-test were used as appropriate to evaluate statistically significant variations between groups of samples. To control the False-Discovery-Rate during multiple comparisons, *p*-values were adjusted using the Benjamini-Hochberg procedure. All tests were two-sided and a *P* < 0.05 was considered statistically significant. Absence of significance was not reported for brevity. Spearmann correlation and linear regression model were used to test the association between parameters.

Statistical analyses were performed using the Sigmaplot software (Systat Software Inc., CA, USA) and RStudio (RStudio: Integrated Development for R. RStudio, Inc., Boston, MA).

## Results

### Leukocyte infiltrate with immunosuppressive features increases from grade II to grade IV gliomas

**Fig. 1:**
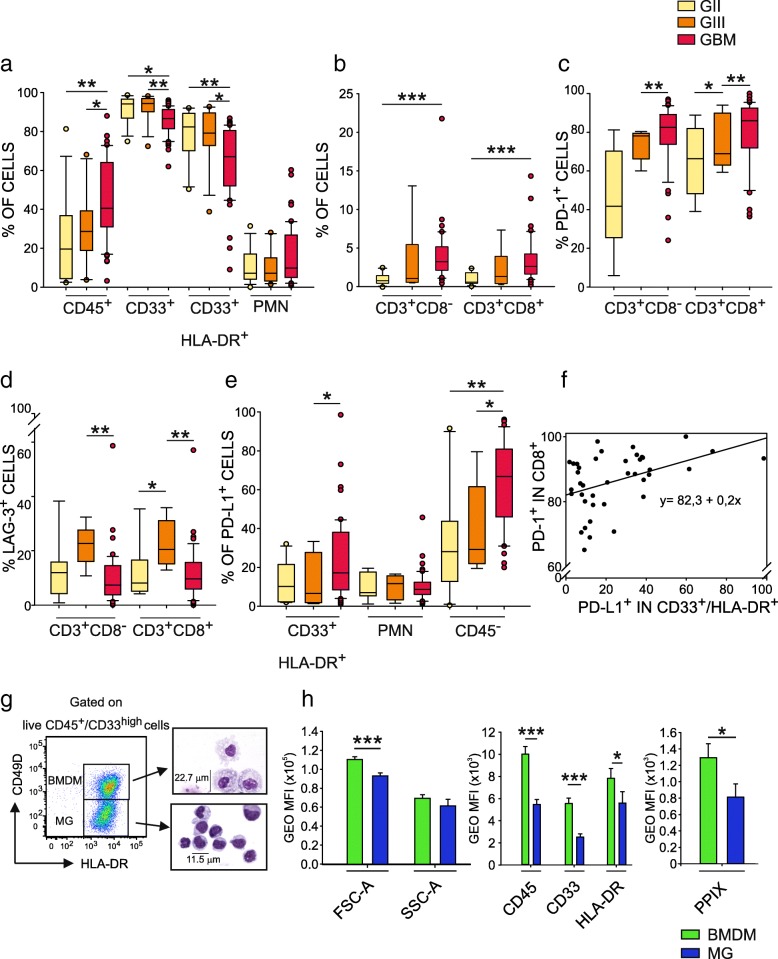
Distribution of tumor-infiltrating leukocytes in gliomas. **a** and **b** Box Plots show the median, 25th and 75th percentile of the frequency of tumor-infiltrating leukocytes, whiskers extend to 1.5 inter-quartile range and outliers are shown by dots. Grade II gliomas are yellow, grade III gliomas orange and GBM red (*n* = 13 for CD45^+^, CD33^+^, CD33^+^/HLA-DR^+^, CD33^dim^/HLA-DR^−^ cells in grade II gliomas, *n* = 12 for grade III gliomas and *n* = 51 in GBM; *n* = 10 for CD3^+^, CD4^+^, CD8^+^ cells in grade II gliomas, *n* = 6 for grade III gliomas and *n* = 46 for GBM patients). CD45^+^ cells were gated among live cells, CD33^+^ cells among CD45^+^ leukocytes, myeloid subsets CD33^+^/HLA-DR^+^ and CD33^dim^/HLA-DR^−^ were gated on CD33^+^ cells, while lymphocytes on CD33^−^/SSC^low^ cells. **c** PD-1 and (**d**) LAG-3 expression in CD3^+^CD8^−^ and CD3^+^CD8^+^ cells in gliomas (*n* = 9 for grade II gliomas, *n* = 5 for grade III gliomas and *n* = 47 for GBM). **e** PD-L1 expression in CD33^+^/HLA-DR^+^ (*n* = 10 for grade II gliomas, *n* = 7 for grade III gliomas and *n* = 50 for GBM), CD33^dim^/HLA-DR^−^ (*n* = 9 for grade II gliomas, *n* = 7 for grade III gliomas and *n* = 50 for GBM), and CD45^−^ cells (*n* = 10 for grade II gliomas, *n* = 7 for grade III gliomas and *n* = 46 for GBM). **f** Linear regression model between PD-L1 expression in CD33^+^/HLA-DR^+^ cells and PD-1 expression in CD8^+^ T cells (*p* = 0,00683). **g** Macrophage subset identification based on CD33^high^, PMN (CD33^int^/SSC^high^ cells) exclusion and CD49D and HLA-DR markers in GBM (left plot). The two populations were purified by FACS sorting and MGG stained (right images). **h** Intensity of morphological parameters (left histogram), CD45, CD33, HLA-DR (middle histogram), and PpIX expression (right histogram; *n* = 23, for CD45 analysis *n* = 14) in BMDM (green) and MG (blue). Mann-Whitney test, *p < 0.05, **p < 0.01, ***p < 0.001, ****p < 0.0001

To evaluate the immune web at the tumor site, we performed a detailed analysis of the leukocyte infiltrate by multicolor flow cytometry (Additional file 1: Table S1) in tumor tissues from untreated grade II, III and IV glioma patients (Table 1), processed immediately after resection. We found a recurrent presence of CD45^+^ leukocytes infiltrating human gliomas, increasing significantly in grade IV gliomas (median 19.6% grade II vs 28.6% grade III vs 40.3% grade IV). Of note, the majority of infiltrating leukocytes consisted of CD33^+^ myeloid cells (91.2, 92.2 and 85.6% in grade II, III and IV gliomas respectively), mainly composed by CD33^+^/HLA-DR^+^ macrophages (mean of 85.2% in grade II, 84.3% in grade III and 64.7% in GBM), and by a lower percentage of CD33^dim^/HLA-DR^−^ polymorphonuclear cells (PMNs, 10.8% in grade II, 10.2% in grade III and 15.8% in GBM) (Fig. 1a). T cells were also present, although in a small amount, but both CD4^+^ (defined as CD3^+^/CD8^−^) and CD8^+^ T cells increased significantly from grade II to grade IV gliomas (Fig. 1b), paralleled by a significant expression of PD-1, increasing from grade II to grade IV (mean of 45.4% vs 73.9% vs 79.0% in grade II, III and IV for CD3^+^CD8^−^ cells, and 64.8% vs 74.9% vs 80.4% in grade II, III and IV for CD3^+^CD8^+^ cells, respectively), as shown in Fig. 1c. Another molecule associated to T cell dysfunction, LAG-3, was present on all T cells infiltrating grade II to IV gliomas, although at lower levels than PD-1, and its expression peaked in grade III gliomas (Fig. 1d).

As far as PD-L1 expression in the glioma microenvironment is concerned, the highest expression was present on CD45^−^ cells, with a progressive and significant increase from grade II to grade IV gliomas, as already reported at transcriptional level by Wang et al. [17]. However, PD-L1 expression was also present on macrophages, in line with previous results [18, 19], with a significant increase in grade IV tumors (Fig. 1e). PMNs show a lower PD-L1 expression as compared to macrophages and CD45^−^ cells and no statistically significant differences were observed. Interestingly, when we considered CD8^+^ T cells expressing PD-1 higher than 60% (39 out of 45 GBM cases), we observed a significant correlation with PD-L1-expressing macrophages (Fig. 1f), but not with PMN and with CD45^−^ cells.

### Identification of a set of markers distinguishing MG from BMDMs

Given the abundance of macrophages in GBM infiltrate, we set out to identify brain resident MG from circulating monocytes that migrate to the tumor site. Until now, an unequivocal phenotypic and functional distinction between these two cell types is missing, and one marker differentially expressed between MG and BMDM is CD49D [6, 20]. We thus began to discriminate macrophages with this marker, in conjunction with CD45, CD33 and HLA-DR. This analysis revealed the presence of two main myeloid cell subsets identified as CD45^+^/CD33^+^/HLA-DR^+^/CD49D^+^, corresponding to BMDM, and CD45^+^/CD33^+^/HLA-DR^+^/CD49D^−^, corresponding to MG cells (Fig. 1g). After cell sorting, morphological evaluation of cytospins of these subsets indicated that the two populations have distinct morphological characteristics; compared to resident MG CD49D^−^ cells, CD49D^+^ BMDM are larger cells (22.7 μm as mean diameter of BMDM vs 11.5 μm of MG), with an abundant and vacuolated cytoplasm, and smaller nucleus-to-cytoplasm ratio, a typical morphology of tissue macrophages (Fig. 1g). These morphological differences were also confirmed by flow cytometry, as BMDMs show a significantly higher forward-scatter (FSC) than MG (Fig. 1h). Moreover, the two macrophage populations had a characteristic phenotype, since BMDMs express HLA-DR, CD45, CD33 markers at higher levels than MG cells (Fig. 1h middle panel), thus permitting their unambiguous identification.

### BMDM infiltration in GBM is responsible for the immunosuppressive gradient stemming from the tumor core

**Fig. 2:**
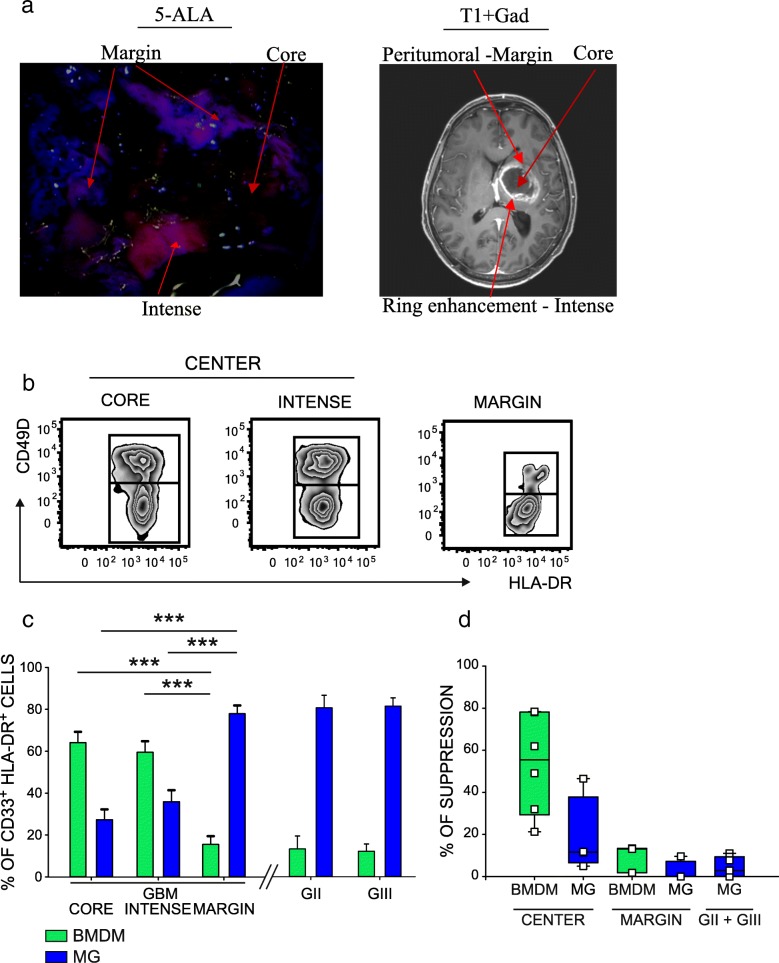
MG and BMDM characterization in different glioma areas and analysis of their immune suppressive activity. **a** Surgical microscopic view under blue light (left panel) and preoperative Magnetic Resonance T1-weighted image after gadolinium administration (right panel) of a patient with a left deep GBM. Different fluorescence intensities are detected in distinct tumor areas: a bright fluorescence corresponds to the ring tumor enhancement at MRI, a dim fluorescence is present in the peritumoral infiltration, lack of fluorescence is in the central necrotic area. **b** Representative flow cytometry panels and (**c**), cumulative data of BMDM (green) and MG (blue) cells in three tumor layers identified by 5-ALA fluorescence in GBM tissues (*n* = 24 core, *n* = 30 intense fluorescence, *n* = 19 marginal samples) (left histograms) and from grade II (*n* = 11) and III (*n* = 9) glioma patients (right plots). **d** Immunosuppressive activity of BMDM (green) and of MG (blue) isolated by FACS sorting from the central intense fluorescence layer, or from the surrounding peritumoral space of GBM patients (*n* = 7 for BMDM in the center, *n* = 4 for MG in the center; *n* = 3 for BMDM in the margin, 4 for MG in the margin). MG tested from grade II (*n* = 2) and grade III (*n* = 3) gliomas. Comparison by Mann-Whitney test, ***p < 0.001

GBM growth follows a multilayer pattern of lesion spread driven by hypoxia and characterized by a central necrotic area [21] and a marginal area [22–25]. We analyzed different layers of the tumor mass to understand if myeloid cell infiltrate differs between the center and the marginal areas. Tissue sampling was performed by 5-ALA assisted surgery combined with MRI-neuronavigation. Following preoperative administration of 5-ALA, fluorescent protoporphyrin IX (PpIX) is synthesized and can be visualized under violet light with different fluorescence intensities, allowing the identification of the central necrotic area (core), corresponding to the inner non fluorescent tissue, an intermediate area, brightly fluorescent (intense), and a marginal area corresponding to a dimly fluorescent tissue (margin), (Fig. 2a). A representative example of this analysis is shown in Fig. 2b, demonstrating the coexistence of BMDM and MG in the lesion, but with different proportions in the center or in the marginal area. Collectively, in 30 patients in which the three matched tissues were individually analyzed, BMDM represented 15.5% +/− 3.9% (mean ± SE) of total macrophages in the margin, but their concentration rose in the center of the lesion, both in the core (64.2 +/− 5.1%) and in the intense fluorescent area (59.5 +/− 5.3%) (Fig. 2c). On the contrary, the presence of MG cells in the GBM microenvironment followed inverse proportions in the center versus the marginal area (27.3% +/− 4.9% in the core, 35.9% +/− 5.5% in the intense fluorescent area and 77.9% +/− 3.9% in the margin, Fig. 2c). We then evaluated the presence of macrophages in grade II and III glioma tissues, in which surgery was performed without 5-ALA. In grade III tumors, resected samples corresponded to enhancing regions at T1-Weighted Images with gadolinium, while grade II gliomas were without contrast enhancement. We observed that the presence of BMDM in grade II and III gliomas was low or absent (mean of 13.7% in grade II and 12.6% in grade III gliomas; Fig. 2c, right histograms), with an infiltration profile similar to that of the marginal area of GBM tissue. IDH status had no impact on the MG/BMDM infiltrate: in 76 GBM analyzed, 4 samples were mutated, but with no significant difference in terms of macrophage composition; the same applies to grade II, in which only one sample out of 13 analyzed was wild type, and to grade III, which harbored 3 wild type samples out of the 12 collected, but no major differences were observed in terms of immune cell composition.

Taken together, these results suggest that BMDM accumulation characterizes grade IV gliomas, and that these cells progressively infiltrate in the lesion from the marginal to the central area. We tested the hypothesis that macrophages possess an immune suppressive activity conditioned not only by the ontogeny, but also by the context of the tumor microenvironment. To this end, we sorted BMDM and MG cells both in the center and in the marginal tumor area, and tested their ability to interfere with the proliferation of activated T cells. Results from these experiments revealed that when BMDMs are located in the core of the lesion, they possess an immunosuppressive activity (range, 21.3–78.4%), which is higher than that exerted by MG cells sorted from the same central part of the lesion (range,4.9–46.5%). In the marginal part of the tumor, instead, both populations showed a reduced suppressive activity (Fig. 2d, left histograms), thus highlighting that BMDMs acquire immune suppressive ability as they migrate to the center of the lesion. We also evaluated the functional activity of sorted MG cells in grade II and III gliomas, the main macrophage population in these tumor tissues, and observed that these cells have a negligible immunosuppressive function in both grade II and in grade III gliomas ranging from 0 to 11% (Fig. 2d, right histogram).

These results highlight that the immunosuppression present in GBM tumor microenvironment depends on the infiltration of BMDM in the central part of tumor mass and that BMDMs and MG have an intrinsic different tolerogenic capability.

### PpIX fluorescence emission: a new tool to identify immunosuppressive macrophages in GBM tissues

**Fig. 3:**
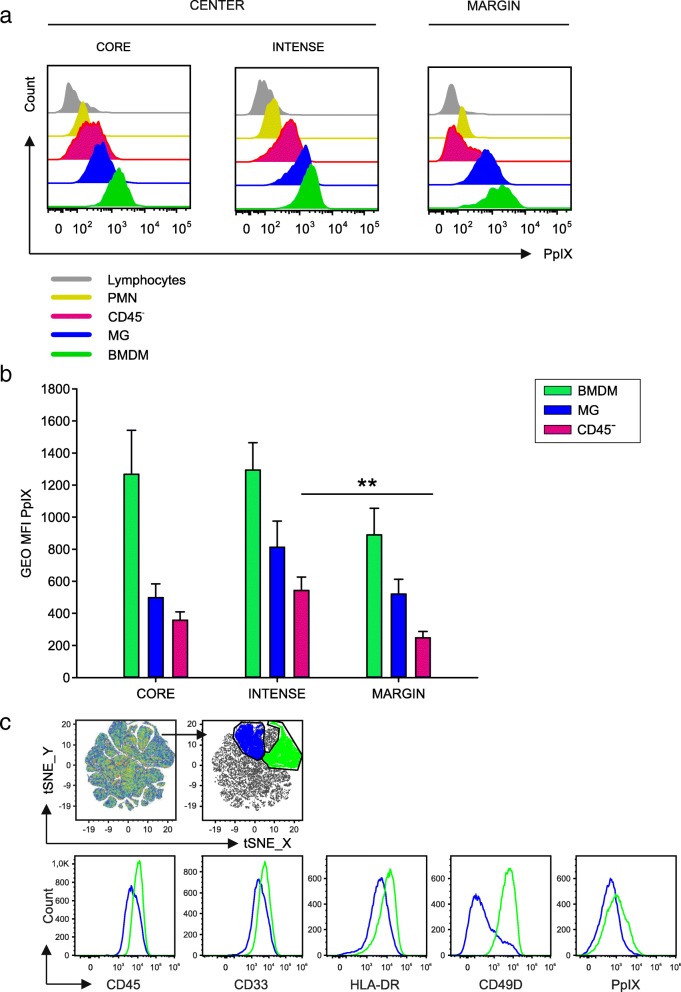
Fluorescence emission of PpIX from cell subsets in the tumor microenvironment. **a** Representative flow cytometry plot and (**b**) cumulative analysis of 5-ALA fluorescence emission by CD45^−^ cells (pink), MG (blue) and BMDM (green) (*n* = 15 core samples, *n* = 21 intense fluorescence samples and *n* = 13 margin samples). **c** t-SNE analysis of live cells infiltrating the intense fluorescence layer (*n* = 11 samples) of GBM patients. Combined analysis on the following parameters: CD45, CD33, HLA-DR, CD49D, PPIX. In the two main clusters obtained after t-SNE analysis (blue and green populations), the expression of the single markers was analyzed (blue and green histograms). Comparison by Mann-Whitney test, **p < 0.01

Taking advantage of 5-ALA administration to GBM patients, we analyzed fluorescence emission of PpIX in tumor tissue by tumor cells, evaluated as CD45^−^ in the center of the lesion, but also from all the leukocyte subsets. Contrary to expectations that 5-ALA is mainly metabolized by tumor cells, we observed that the strongest emission of fluorescence was from the macrophages in all three layers, with different intensity between MG and BMDM in each layer (Fig. 3 a and b); the fluorescence of PpIX from BMDMs was always brighter than that of MG cells. PpIX emission can thus be used as a cell marker capable of discriminating between the two populations (Fig. 1h, right panel) along with morphological markers (FSC and SSC, Fig. 1h, left panel) and with CD45, CD33, HLA-DR (Fig. 1g, middle histograms). To evaluate if the combination of the previously described markers could lead to the unambiguous discrimination of BMDM and MG, we performed an unsupervised T-Distributed Stochastic Neighbor Embedding (t-SNE) analysis by combining all these parameters, and we obtained the clusterization of live cells present at the tumor site (Fig. 3c, first left plot). By gating on the two main clusters (Fig. 3c, blue and green area in the upper part) and analyzing the expression of the single markers, we could clearly identify the phenotype of MG and BMDM, thus reinforcing our results on the identification of macrophage subsets with this marker combination (Fig. 3c).

**Fig. 4:**
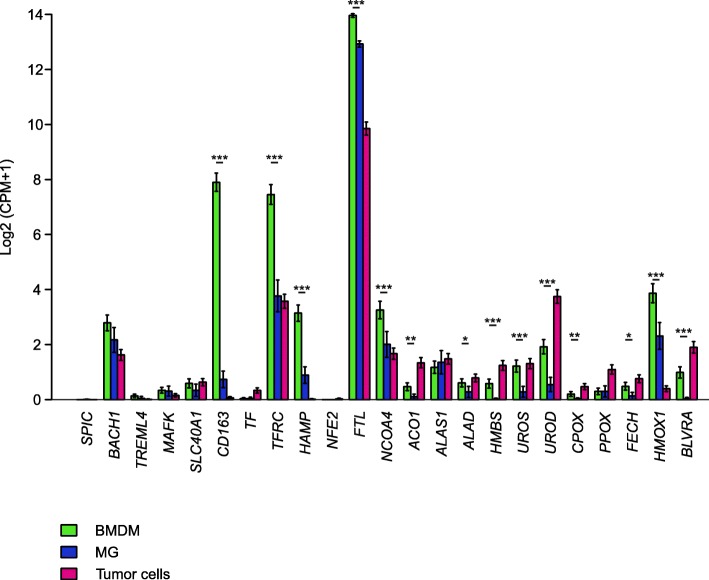
Gene expression analysis of genes involved in heme metabolism in BMDM (green), MG (blue) and tumor cells (pink). Expression of heme-iron metabolism genes obtained from scRNAseq of neoplastic and immune cells from primary gliomas. Both whole tumor and CD11b-purified single-cells suspensions were subjected to scRNA-seq. Bars denote mean expression and whiskers denote the standard error of the mean. Significance assessed via a t-test, *p < 0.05, **p < 0.01, ***p < 0.001

The progressive increase in the accumulation of 5-ALA-induced PpIX in BMDM from the marginal to the central area suggests not only that iron metabolism is sustained in myeloid-infiltrating cells, but also that it is higher than that of tumor cells. To evaluate the role of iron metabolism in the tumor microenvironment of GBM, we analyzed an external data set of eight cases of GBM that were profiled following CD11b selection either via magnetic beads or immunopanning and subjected to single-cell RNA sequencing [10]. Classification of macrophage lineage and of tumor cells was performed using previously described gene signature [10]. Analysis of the expression of the genes implicated in iron metabolism in BMDM, MG and tumor cells showed a significant overexpression of many of these genes in BMDM, compared to MG cells (Fig. 4). In fact, genes involved in iron uptake (CD163 and TFRC), storage (FTL, HAMP, ACO1 and NCOA4), metabolism (FECH, UROS, UROD, HMBS, CPOX and ALAD), and catabolism (BLVRA and HMOX1) are all overexpressed in BMDM (Fig. 4). Collectively, these results highlight that BMDM possess a sustained iron-recycling metabolism.

### BMDM are target of preferential incorporation by lipid nanocapsules in the tumor microenvironment of grade IV gliomas

**Fig. 5:**
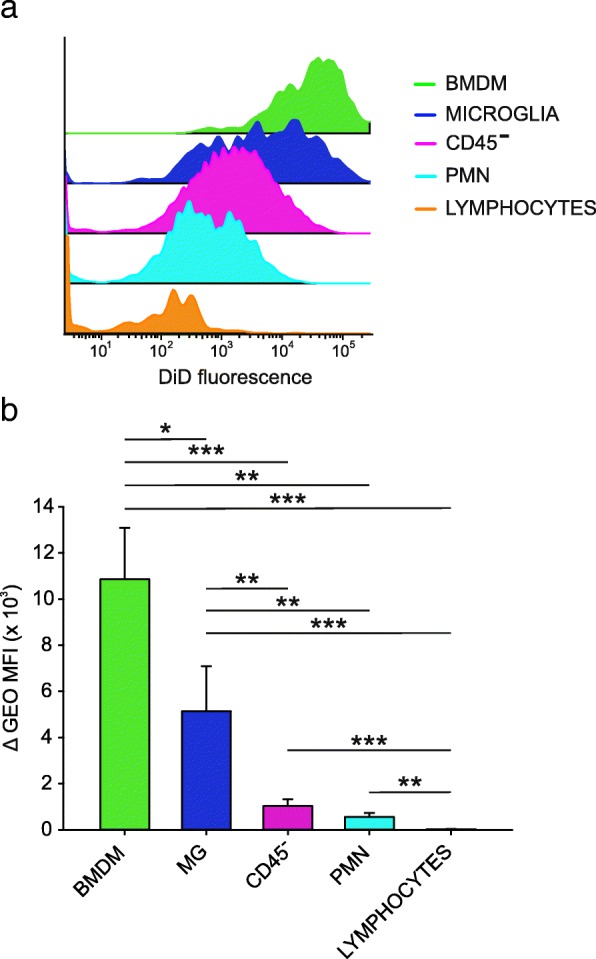
Uptake of LNCs loaded with DiD by cell subsets in GBM. **a** Representative example showing DiD fluorescence intensity of different leukocyte populations and CD45^−^ tumor cells after over-night incubation with DiD-loaded LNCs of cell suspensions. **b** Fluorescence intensities of DiD-loaded LNCs and blank-LNCs in different GBM subsets. *N* = 9 for BMDM, MG, CD45^−^ tumor cells and lymphocytes; *n* = 6 for PMN. Mean and SE are reported; Mann-Whitney U test *p < 0.05, **p < 0.01, ***p < 0.001

We previously demonstrated that lipid nanocapsules (LNCs) loaded with a cytotoxic drug efficiently target immune suppressive monocytic cells in the blood of melanoma patients [26]. We thus evaluated the incorporation of fluorochrome-labeled DiD LNCs by the cell suspensions obtained after dissociation of the central area of the GBM tissue with intense PpIX fluorescence, in which immune suppressive myeloid cells are abundant. Results indicate that lymphocytes, PMN and CD45^−^ cells have a low internalization capability, while DiD fluorescence is increased in macrophage populations, in particular in BMDMs, that show significantly higher levels of uptake as compared to MG. The immunosuppressive BMDM population is therefore the preferential target of this nanocarrier system. (Fig. 5 a, b).

### BMDM infiltration in GBM tissues is sustained by circulating monocytes

**Fig. 6:**
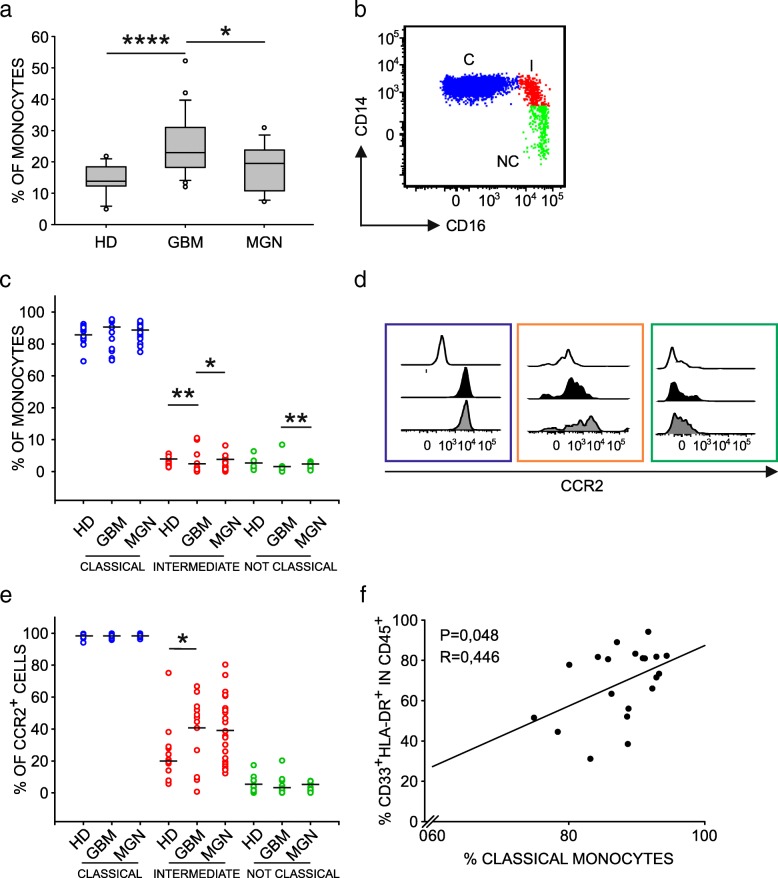
Characterization of monocyte dysregulation in GBM patients. **a** Boxplots of the distribution of percentage of monocytes in blood samples from HD (*n* = 12), GBM (*n* = 24) and meningioma (MNG) (*n* = 13) patients, calculated as HLA-DR^+^ cells among PBMCs. **b** Analysis of monocyte subsets in whole blood using CD14 and CD16 markers. Dot plot gated on PBMCs shows classical monocytes (C) CD14^high^/ CD16^−^ and intermediate subset (I) and a non-classical subset (NC). (**c**) Distribution of C, I and NC monocytes in meningioma (MNG, *n* = 13) and glioblastoma patients (GBM, *n* = 24) in comparison to HDs (*n* = 12). **d** Representative example of CCR2 expression on monocyte subsets in GBM patients. White histograms show fluorescence minus one (FMO) controls, black histograms refer to MFI values of CCR2^+^ cells of healthy donor, while grey histograms indicate MFI values of CCR2^+^ cells among C (blue), I (orange), and NC (green) monocytes of GBM patients. **e** Cumulative data showing CCR2 expression in MNG (*n* = 13) and GBM patients (*n* = 24) compared to HDs (*n* = 12). Mann-Whitney test for statistical significance between pairwise groups. **f** Correlation between the percentage of classical monocytes in PBMCs and that of macrophages among GBM-infiltrating leukocytes. Spearman’s rank-order correlation on 20 paired samples

Since immunosuppressive macrophages that infiltrate GBM are blood-derived, we investigated the characteristics of circulating monocytic cells in the same patients and, as previously reported [7, 27], observed a higher percentage of circulating monocytes compared to a group of age and gender matched healthy donors (HD) (Fig. 6a). As an additional comparison, we also took into account a group of patients with WHO grade I and II meningioma (Table 1) and observed a significantly higher percentage of monocytes in GBM patients only (Fig. 6a). Given that preoperative steroids are administered to both meningioma and GBM patients, this result indicates that monocyte alterations strictly depend on tumor type, and rule out the contribution of steroid treatment.

We next evaluated in detail the composition of blood monocytes to discriminate the three main subsets that are classical monocytes (C=CD14^+^/CD16^−^), intermediate (I=CD14^+^/CD16^+^) and non-classical subsets (NC=CD14^−^/CD16^+^) (Fig. 6b), and further analyzed the expression of CCR2 on their surface (Fig. 6d), as it is known that CCL2 chemokine promotes monocyte accumulation at the tumor site, and has already been implicated in the recruitment of myeloid cells in glioma [28, 29]. Results indicate a decrease of intermediate monocytes (Fig. 6c) but a significant increase of CCR2^+^ cells among the same subset (Fig. 6e), thus suggesting that this population is actively recruited at tumor site. We then tested the presence of a significant association between the levels of circulating and tumor-infiltrating myeloid cells and observed a positive correlation between the percentage of circulating classical monocytes and that of macrophages at tumor site (Fig. 6f), in line with the current hypothesis that classical monocytes are the first to be recruited from the bone marrow and have the potential to give rise to intermediate monocytes first and later to non-classical monocytes [30].

## Discussion

Recently, the immune microenvironment in gliomas has been the subject of intense research. A great impulse has been given by deconvolution studies, which, with the use of a bioinformatic approach, showed that MG and BMDM possess a different transcriptional program [10, 31]. Our work extends previous studies as follows: first, we were able to isolate and test the immune suppressive ability of BMDM and MG, going beyond the transcriptional profile and testing their effective immunoregulatory function; second, by exploiting an imaging surgical technique, we documented the regional contribution to immune suppression of each macrophage cell subset, and third, we demonstrated that isolated microglia cells from grade II and III gliomas lack immunosuppressive activity. Collectively, these results indicate that macrophages’ tolerogenic properties in GBM depend not only by ontogeny, but also by the regional distribution in the tumor area.

The characterization of BMDM and MG after cell sorting revealed that blood-derived macrophages were bigger in size and were more complex than their resident counterparts (Fig. 1 g and h), a morphological feature in line with the presence of what appears to be remnants of ingested cells, revealing different phagocytic capacities as highlighted by LNC incorporation. Based on previous microscopic and morphological studies, both BMDM and MG were considered activated cells in a growing brain tumor [32–34]. However, our results demonstrate that such cells differ, at least as far as their immunosuppressive activity and phagocytic activity is concerned. In fact, resident macrophages participate only marginally in the phenomenon of immune suppression, and this aspect suggests that blood monocytes recruited to the tumor are already committed to a program of immune suppression. However, they only acquire full immune suppressive ability in the center of the lesion, and not in the surrounding region. In this regard the concept of “heritage” carried by these cells, due to their different origin and identified by the CD49D marker, is of translational importance because it shows that BMDMs are endowed with a high immunosuppressive potential, a trait that renders them particularly harmful for patient outcomes, but at the same time it also highlights a potential target of intervention to block their activity and/or recruitment.

Local immunosuppression in GBM also extends to the presence of checkpoint inhibitors on T cells and to their ligands on tumor cells and on innate immune cells, thus showing that multiple mechanisms contribute to the immune suppressive microenvironment in these patients. Interestingly, these checkpoints show lower expression in grade II and III gliomas, in line with previous results on PD-L1 [17, 35], but our results also expanded the analysis on the T cell counterpart. The functional association in GBM of a small, but recurrent population of T cells, bearing a high PD-1 expression, and of its ligand on macrophages (Fig. 1f), suggests that this axis plays a significant role in the suppression exerted by tumor macrophages. Given the low number of IDH mutant patients among the GBM group and vice versa for grade II and III gliomas, PD-L1 and PD-1 distribution segregates with patients IDH status, as previously reported [35].

The presence of a recurrent immunosuppressive infiltrate in grade IV gliomas should be taken into account in new clinical studies of immunotherapy, as a large clinical trial with immune checkpoint inhibitors did not prove its efficacy [36]. Moreover, despite the possibility to induce neoantigen-specific T cells in vaccinated GBM patients, capable to successfully traffic to the tumor site, such T cells are insufficient to induce clinically relevant responses [37, 38]. Thus, blocking the immune suppression in these patients appears a necessary step to stimulate an efficient anti-tumor immune response, and targeting the myeloid infiltrate in GBM represents a new therapeutic strategy for future clinical studies, in combination with immune stimulation.

Our data show that the functional differences between MG and BMDM extend beyond the immune suppression task, since their iron-related metabolism also shows significant differences in these macrophages. Iron metabolism is an important hallmark for macrophages, and it is not surprising that blood-derived cells maintain such characteristics when recruited to the tumor lesion. Moreover, several studies suggest that tumor cells exploit this macrophage ability to supply iron to the tumor [39, 40]. In fact, in response to inflammatory conditions, macrophages increase the iron storage, while in the tumor microenvironment they release iron, which is required to sustain tumor survival and growth [40]. Whether the iron metabolism is linked to the program of immune suppression remains to be explored, but our data open up the possibility of targeting this circuit with drugs interfering with this pathway.

The alteration of the myeloid compartment in GBM patients is also confirmed by the decrease of circulating CD16^+^ intermediate monocytes showing an increase of CCR2 expression. It is currently believed that classical monocytes are the first cell subset to transit from the bone marrow to the circulation, and they have the potential to give rise to intermediate monocytes, and later to non-classical monocytes [30]. Therefore, the reduction in intermediate monocytes present in GBM patients, coupled with the simultaneous increase in CCR2 expression in this cell subset, suggests the possibility that CD14^+^/CD16^+^ monocytes represent the cell subset actively recruited to the tumor in GBM patients. This hypothesis is in line with the positive correlation between the percentage of classical monocytes present in the peripheral blood of GBM patients and the percentage of macrophages in the tumor (Fig. 6f), thus suggesting an active process of monocyte recruitment dictated by GBM milieu, in line with murine fate mapping studies [6]. It thus appears that circulating myeloid cells also have a critical role in GBM, as they are the source sustaining the accumulation in the lesion and therefore blocking macrophage recruitment to the tumor might represent another strategy to limit tumor growth.

## Conclusions

In this study, we demonstrate the presence of an extensive immunosuppressive microenvironment in GBM, but not in grade II and III gliomas, due to the presence of blood-derived macrophages, expressing PD-L1, and of T cells showing markers associated to impaired T cell function. Our study shows that macrophages of bone marrow origin migrate to the tumor site and accumulate in the central area of GBM, exerting a strong immune suppression, while resident microglia exerts low or no immunosuppressive function. Microglia constitutes the majority of macrophages in grade II and III gliomas and is devoid of significant immunosuppressive activity. Besides the tolerogenic properties, differences exists between resident versus blood-derived macrophages, such as iron metabolism and ability to efficiently internalize a nanocarrier system that could be used to target them at tumor site.

## Additional file

Additional file 1: **Table S1.** Cell populations analyzed in tumor tissues of glioma patients. Supplementary materials and methods. Description of patient characteristics, multiparametric flow cytometry, functional assay, t-SNE analysis and experiments with nanoparticles. (DOCX 22 kb)

## Abbreviations

5-ALA: 5-aminolevulinic acid
ACO1: Aconitase 1
ALAD: Aminolevulinate Dehydratase
BLVRA: Biliverdin Reductase A
BMDM: bone marrow-derived macrophages
CCL2: C-C Motif Chemokine Ligand 2
CCR2: C-C chemokine receptor type 2
CNS: central nervous system
CPOX: coproporphyrinogen oxidase
FACS: Fluorescence-Activated Cell Sorting
FECH: Ferrochelatase
FLAIR: Fluid Attenuated Inversion Recovery
FMO: Fluorescence Minus One
FSC: Forward scatter
FTL: Ferritin light chain
GBM: Glioblastoma
HAMP: Hepcidin Antimicrobial Peptide
HD: Healthy donor
HMBS: Hydroxymethylbilane Synthase
HMOX1: Heme Oxygenase 1
IDH: Isocitrate dehydrogenase
LAG-3: lymphocyte-activation gene 3
LNC: lipid nanocapsules
MFI: mean fluorescence intensity
MG: microglia
MGG: May-Grunwald Giemsa
MNG: meningioma
MRI: Magnetic Resonance Imaging
NCOA4: Nuclear Receptor Coactivator 4
OS: Overall Survival
PBMC: Peripheral blood mononuclear cell
PD-1: Programmed cell death protein 1
PD-L1: Programmed death-ligand 1
PMN: Polymorphonuclear cells
PpIX: Protoporphyrin IX
scRNAseq: Single-cell RNA sequencing
SSC: Side-scatter
TFRC: Transferrin receptor protein 1
t-SNE: T-Distributed Stochastic Neighbor Embedding
UROD: Uroporphyrinogen Decarboxylase
UROS: Uroporphyrinogen III Synthase
WHO: World Health Organization
