# Supplementary material for: The immune suppressive microenvironment of human gliomas depends on the accumulation of bone marrow-derived macrophages in the center of the lesion
Source: J Immunother Cancer. 2019 Feb 27;7:58. doi: 10.1186/s40425-019-0536-x (PMC6391795; doi:10.1186/s40425-019-0536-x)
Supplement: Supplementary file 1 — Table S1. Cell populations analyzed in tumor tissues of glioma patients. Supplementary materials and methods. Description of patient characteristics, multiparametric flow cytometry, functional assay, t-SNE analysis and experiments with nanoparticles. (DOCX 22 kb) [file 40425_2019_536_MOESM1_ESM.docx]

**Additional files:**

**Additional file 1:**

**Table S1**. Cell populations analyzed in tumor tissues of glioma patients

**Supplementary Materials and methods.** Description of patients’characteristics, multiparametric flow cytometry, functional assay, t-SNE analysis and experiments with nanoparticles.

**Supplementary Table S1. Cell subsets identified in tumor lesions of glioma patients.**

|  |  |
| --- | --- |
|  |  |
| **Lineage** | **Immunophenotype** |
|  |  |
|  |  |
| MG^a^ | CD45^+^ CD33^+^ HLA-DR^+^ CD49d^-^ PpIX^int^ |
| BMDM^b^ | CD45^+^ CD33^+^ HLA-DR^+^ CD49d^+^ PpIX^high^ |
| PMN^c^ | CD45^+^ CD33^int^ HLA-DR^-^ CD15^+^ |
| T cells | CD45^+^ CD33^-^ CD3^+^ LAG-3^+^ |
|  | CD45^+^ CD33^-^ CD3^+^ PD-1^+^ |
| CD8^+^ T cells | CD45^+^ CD33^-^ CD3^+^ CD8^+^ LAG-3^+^ |
|  | CD45^+^ CD33^-^ CD3^+^ CD8^+^ PD-1^+^ |
| CD3^+^ CD8^-^ T cells | CD45^+^ CD33^-^ CD3^+^ CD8^-^ LAG-3^+^ |
|  | CD45^+^ CD33^-^ CD3^+^ CD8^-^ PD-1^+^ |
| PD-L1^+^ myeloid subsets | CD45^+^ CD33^+^ PD-L1^+^ |
|  | CD45^+^ CD33^+^ HLA-DR^+^ PD-L1^+^ |
|  | CD45^+^ CD33^int^ HLA-DR^-^ PD-L1^+^ |
|  | ~~CD45~~^~~+~~^ ~~CD33~~^~~+~~^ ~~HLA-DR~~^~~low~~^ ~~PD-L1~~^~~+~~^ |
|  |  |
|  |  |

^a^MG: microglia

^b^BMDM: bone marrow- derived macrophages

^c^PMN: polymorphonuclear cells

**Supplementary Materials and Methods**

**Patient characteristics**

Patients were recruited at the Department of Neurosurgery, Padova University Hospital, Italy. We obtained peripheral blood and freshly resected tumor material from 76 patients with confirmed cases of GBM. Eleven of the 76 patients (corresponding to 14 biopsies) were recurrent GBM and were only included in the phenotypical analysis of tumor lesion (Table 1). For some patients, multiple biopsies were analyzed, corresponding to different layers of tumor area, as defined by 5-ALA emission, for a total of 113 tumor samples. Tumor biopsies were also analyzed from 13 grade II glioma patients and 12 grade III glioma patients. Peripheral blood was obtained from 13 meningioma patients, corresponding to 8 grade I and 5 grade II cases (Table 1). As controls, peripheral blood of 35 healthy donors, matched for age and sex, was analyzed.

**Multiparametric flow cytometry**

To analyze classical, intermediate and non-classical monocytes in the peripheral blood of HDs, meningioma and GBM patients we used an antibody mixture containing anti-CCR2 FITC (Miltenyi Biotec), anti-CD14 APC-H7 (BD Biosciences), anti-CD16 PerCP-Cy5.5 (BioLegend), anti-HLA-DR APC (BD Biosciences). ~~.~~

Cell suspension obtained from glioma biopsies after enzymatic digestion was labelled with different antibody mixtures optimized to characterize myeloid and lymphocyte cell populations. 5x10^5^ cells were washed with PBS plus 1% FBS and incubated for 10 minutes at 4°C with Fc-Receptor Blocking reagent (Miltenyi Biotec). All other mAbs were then added and incubated for 20 minutes at 4°C. Subsequently, cells were washed with PBS plus 1% FBS and samples were acquired by flow cytometer. The antibody mixtures used to analyze tumor biopsies contained LIVE/DEAD Fixable Aqua (Life Techonologies), LIVE/DEAD Near IR (Life Technologies), anti-CD45 BV421 (BD Biosciences), anti-CD45 FITC (Miltenyi Biotec), anti-CD33 PE-Cy7 (eBioscience) or anti-CD33 APC (BD Biosciences), anti-HLA-DR APC (BD Biosciences), Lin cocktail 1 FITC (BD Biosciences), anti-CD11b Alexa700 (BD Pharmingen), anti-PD-L1 PE (eBioscience), anti-CD14 APC-H7 (BD Biosciences), anti-CD3 PE-Cy7 (Beckman Coulter), anti-CD8 APC-H7 (BD Biosciences), anti-LAG-3 FITC (AdipoGen), anti-PD1 PE (MiltenyiBiotec), anti-CD49D PE (BioLegend). After incubation with Did-loaded LNCs, cells obtained from GBM biopsies were labelled with LIVE/DEAD Fixable Aqua (Life Techonologies), anti-CD45 BV421 (BD Biosciences), anti-CD14 FITC (Miltenyi Biotec), anti-HLA-DR PerCP-Cy 5.5 (BioLegend), anti-CD49D PE (BioLegend).

Data were acquired using a LSRII flow cytometer (BD Biosciences) equipped with 4 lasers (405nm, 488nm, 561nm, 640nm) and analysis was performed by FlowJo software (Three Star Inc). Fluorescence minus one (FMO) controls for HLA-DR, PD-L1, PD-1 and LAG-3 were used as negative controls. The fluorescence of PpIX was detected by 635 long-pass filter coupled to a 670/30 filter, after excitation by 405 nm laser. Sample autofluorescence was set on the vague fluorescent cells.

All antibodies used for flow cytometry were titrated in a lot-dependent manner.

**Isolation of myeloid cell subsets and test of immunosuppressive activity**

To separate CD49D^+^/HLA-DR^+^ cells and CD49D^-^/HLA-DR^+^ cells present in the tumor, cell suspension obtained after enzymatic digestion of glioma biopsies was stained with Livedead Aqua (Invitrogen), anti-CD45 BV421 (BD Biosciences), anti-CD49D PE (BioLegend) and anti-HLA-DR APC (BD Biosciences) and filtered through a 100 μM cell strainer. If any debris was present in digested samples before staining, Debris Removal Solution (Miltenyi Biotec) was used to remove them, following manufacturer’s instructions. CD49D^+^/HLA-DR^+^ or CD49D^-^/HLA-DR^+^ cell subsets were then separated by FACS sorting (BD FACS ARIA III). The purity of each fraction was >90%. For some patients we received three tumor samples, corresponding to different tumor layers, and we separated the same populations described above (CD49D^+^/HLA-DR^+^ and CD49D^-^/HLA-DR^+^ cells) from each tumor sample by FACS sorting.

Immunosuppressive activity of myeloid cells isolated from tumor of glioma patients was performed as previously described [16]. Briefly, PBMCs were stained with 0.5 μM CellTrace™ Violet Cell Proliferation Kit (Invitrogen, Molecular Probes, MA, USA), and activated with coated 1 μg/ml anti-CD3 and 5 μg/ml soluble anti-CD28 (BioLegend, CA, USA). Myeloid cells separated from peripheral blood or tumor tissue of glioma patients were co-cultured at 1:1 ratio with CellTrace-labelled PBMCs in flat bottom 96 or 384 well plates. Cell cultures were incubated for four days at 37°C and 5% CO_2_ in arginine free-RPMI (Biological Industries, Kibbutz Beit Haemek, Israel), supplemented with 150 μM arginine and 10% FBS (SIGMA-Aldrich), 10 U/ml penicillin and streptomycin, and HEPES . At the end of cell culture, cells were harvested, stained with anti-CD3 PE-Cy7 (Beckman Coulter) and analyzed by flow cytometry. Proliferation of T cells was evaluated by assessing the signal of CellTrace on CD3^+^ cells, and considering as proliferating the cells present from generation 3 onwards, or calculating the absolute number of CD3^+^ cells in each sample by TruCount^TM^ tubes (BD Biosciences). In both cases data were normalized assuming the proliferation of T cells cultured alone as 100%.

**Analysis of LNC uptake by tumor-infiltrating leukocytes**

Cell suspension obtained after enzymatic digestion of GBM tissue was diluted to 10^6^ cells/ml in RPMI medium supplemented with 10% FBS, 10 U/ml penicillin and streptomycin, and 0.01 M HEPES, 0.55 mM Arginine (Sigma-Aldrich), 0.24 mM Asparagine (Sigma-Aldrich) and 1.5 mM Glutamine (Sigma-Aldrich). 100 nm positively charged LNCs, loaded with DiD or blank, were added to cell suspension for a final DiD concentration of 50 ng/ml (and to a corresponding LNC concentration for blank LNCs) and left in incubation over-night at 37°C and 5% CO_2_. At the end of incubation, cells were washed and stained for flow-cytometry acquisition and subsequently analyzed by Flowjo software.

**t-SNE analysis:**

To perform t-SNE analysis, we created one concatenated file for 11 intense fluorescence samples, after the reduction of live cell number to 2x10^4^. In the concatenated file, we ran t-SNE among live cells using the following parameters: FSC-A, SSC-A, CD45, HLA-DR, CD33, CD49D, and PpIX and then we analyzed the expression of the single markers in the two main clusters thus identifying BMDM and MG. Flowjo software, version 10.4.1 was used for the analysis.
